# Piscichuvirus-Associated Severe Meningoencephalomyelitis in Aquatic Turtles, United States, 2009–2021

**DOI:** 10.3201/eid3002.231142

**Published:** 2024-02

**Authors:** Weerapong Laovechprasit, Kelsey T. Young, Brian A. Stacy, Steven B. Tillis, Robert J. Ossiboff, Jordan A. Vann, Kuttichantran Subramaniam, Dalen W. Agnew, Elizabeth W. Howerth, Jian Zhang, Shayna Whitaker, Alicia Walker, Andrew M. Orgill, Lyndsey N. Howell, Donna J. Shaver, Kyle Donnelly, Allen M. Foley, James B. Stanton

**Affiliations:** University of Georgia, Athens, Georgia, USA (W. Laovechprasit, K.T. Young, E.W. Howerth, J. Zhang, J.B. Stanton);; National Oceanic and Atmospheric Administration, Pascagoula, Mississippi, USA (B.A. Stacy, L.N. Howell);; University of Florida, Gainesville, Florida, USA (S.B. Tillis, R.J. Ossiboff, J.A. Vann, K. Subramaniam);; Michigan State University, Lansing, Michigan, USA (D.W. Agnew);; Amos Rehabilitation Keep at University of Texas Marine Science Institute, Port Aransas, Texas, USA (S. Whitaker, A. Walker, A.M. Orgill);; National Park Service at Padre Island National Seashore, Corpus Christi, Texas, USA (D.J. Shaver);; Brevard Zoo and Sea Turtle Healing Center, Melbourne, Florida, USA (K. Donnelly);; Florida Fish and Wildlife Conservation Commission, Jacksonville, Florida, USA (A.M. Foley)

**Keywords:** Piscichuvirus, turtles, metagenomics, encephalomyelitis, meningoencephalomyelitis, infectious diseases, viruses, United States, meningitis/encephalitis

## Abstract

Viruses from a new species of piscichuvirus were strongly associated with severe lymphocytic meningoencephalomyelitis in several free-ranging aquatic turtles from 3 coastal US states during 2009–2021. Sequencing identified 2 variants (freshwater turtle neural virus 1 [FTuNV1] and sea turtle neural virus 1 [STuNV1]) of the new piscichuvirus species in 3 turtles of 3 species. In situ hybridization localized viral mRNA to the inflamed region of the central nervous system in all 3 sequenced isolates and in 2 of 3 additional nonsequenced isolates. All 3 sequenced isolates phylogenetically clustered with other vertebrate chuvirids within the genus *Piscichuvirus*. FTuNV1 and STuNV1 shared ≈92% pairwise amino acid identity of the large protein, which narrowly places them within the same novel species. The in situ association of the piscichuviruses in 5 of 6 turtles (representing 3 genera) with lymphocytic meningoencephalomyelitis suggests that piscichuviruses are a likely cause of lymphocytic meningoencephalomyelitis in freshwater and marine turtles.

Wild populations of aquatic turtles are imperiled because of anthropogenic activities (e.g., consumption, collection, fisheries bycatch) (*1*); more than half (186/357) of the recognized species of aquatic turtles in the world are designated as critically endangered, endangered, or vulnerable (*2*). In addition to anthropogenic threats, infectious agents also negatively affect free-ranging turtles. For example, chelonid herpesvirus 5 (*Scutavirus chelonidalpha5*: *Alphaherpesvirinae*) is associated with transmissible fibropapillomatosis in sea turtles around the world (*3*), and epizootic outbreaks of meningoencephalitis in Florida freshwater turtles have been attributed to the recently discovered turtle fraservirus 1 (*Fraservirus testudinis*: *Tosoviridae*) (*4*). However, learning about infectious agents in such turtles is complicated by their aquatic nature and cryptic lifestyles, which prevents full appreciation of the threat posed by viruses to free-ranging turtles.

Recently, numerous viruses in captive and free-ranging nondomesticated animals have been identified. Among the newly discovered viruses, chuvirids (class Monjiviricetes, order Jingchuvirales, family Chuviridae) (*5*) ​are of particular interest. First, they have a broad host range (e.g., phototrophs, a wide array of invertebrates, and vertebrates [fish and snakes]) (*5*–*10*). Second, the genomic structure of viruses in that family is unusual (*11*,*12*). Although other jingchuvirals have nonsegmented linear genomes, chuvirids have been reported to have circular segmented, circular nonsegmented, linear segmented, and linear nonsegmented genomes (*7*,*12*). Phylogenetic analysis of the large (L) protein suggests that jingchuvirals have a unique history among viruses in the class Monjiviricetes (*6*,*12*). Although recent discoveries of chuvirids and their varying genomic structures draw interest from a virologic standpoint, the pathogenicity of chuvirids and jingchuvirals has not been confirmed (*12*). To our knowledge, only 1 published study has demonstrated any jingchuviral from an ill animal: a piscichuvirus (Herr Frank virus 1 [HFrV1]; *Chuviridae*: *Piscichuvirus franki*) in clinically ill snakes (3 of 4 boa constrictors [*Boa constrictor constrictor*] with boid inclusion body disease; all 4 boas were positive for reptarenaviruses) (*13*). However, because no piscichuviral in situ studies were performed with those snakes and meningoencephalitis was not reported, clinical significance of chuvirids remains unresolved. 

To identify the potential cause of lymphocytic meningoencephalomyelitis in several aquatic turtles, we randomly sequenced central nervous system (CNS) tissues from 3 affected turtles and performed in situ hybridization (ISH) on CNS tissues of 6 turtles. We obtained complete genomes for 3 isolates, providing phylogenetic classification and in silico identification of conserved secondary structures at the genome termini and a hypothetical fourth open reading frame (ORF). The relative dissimilarity between the freshwater and sea turtle piscichuviruses raises questions regarding jingchuviral speciation criteria. 

## Materials and Methods

### Samples and Pathologic Examination

**Figure 1:**
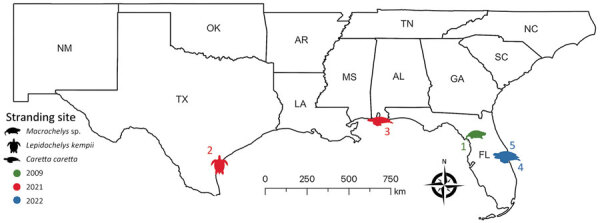
Geographic location and year found for 5 stranded piscichuvirus-infected aquatic turtles with meningoencephalomyelitis, United States, 2009–2021.

We examined isolates from 6 aquatic turtles with idiopathic CNS inflammation (meningoencephalitis to meningoencephalomyelitis): 1 freshwater (alligator snapping turtle, morphologically consistent with either *Macrochelys temminckii* or *M. apalachicolae*) and 5 marine (1 Kemp’s ridley [*Lepidochelys kempii*] and 4 loggerhead [*Caretta caretta*]), by using sequencing (turtles 1–3), ISH (turtles 1–6), or both (Table 1; Figure 1). We used 6 additional retrospective cases as controls: 2 loggerhead turtles with spirorchiid-induced mononuclear meningoencephalitis, 1 Kemp’s ridley turtle with bacterial encephalitis, and 3 turtles lacking meningoencephalomyelitis (1 of each species).

**Table 1:** Biographical information and results summary of 5 turtles positive for FTuNV1 or STuNV1, United States, 2009–2021*

Animal no., common name (taxonomic name)	Sex	SCL, cm	Life stage†	Stranding location‡		Clinical signs	Sequencing	ISH (FTuNV1/ STuNV1)
				Latitude, °N	Longitude, °W			
1, Alligator snapping turtle (*Macrochelys* sp.)	M	56.5	M	29.524879	82.300594	Weak and lethargic	FTuNV1	(+/−)
2, Kemp’s ridley turtle (*Lepidochelys* *kempii*)	F	60.3	I	27.67338	97.16880	Persistent circling and asymmetric buoyancy	STuNV1	(–/+)
3, Loggerhead turtle (*Caretta* *caretta*)	M	94.0	M	30.230591	87.910237	Unresponsive	STuNV1	(ND/+)
4, Loggerhead turtle (*C*. *caretta*)	F	81.8	M	28.04815	80.57892	Weak	ND	(ND/+)
5, Loggerhead turtle (*C*. *caretta*)	F	83.7	I	28.20726	80.65725	Head tremor and cervical ventroflexion	ND	(ND/+)

*FTuNV1, freshwater turtle neural virus 1; I, immature; ISH, in situ hybiridization; M, mature; ND, not done; SCL, straight carapace length; STuNV1, sea turtle neural virus 1. †Life stage estimation was based on the maturation of the reproductive system. ‡Latitude and longitude values correspond to the stranding locations as shown in Figure 1.

All turtles died spontaneously or were euthanized via pentobarbital overdose if their illness was advanced. Gross necropsy included systematic evaluation of all organ systems. We determined sexual maturity by evaluating the reproductive system. We aseptically collected fresh tissue samples, including from the brain and spinal cord (turtle 1–3), and stored them at –80°C. We preserved samples in neutral-buffered 10% formalin fixative for 24–48 hours before processing for histopathologic analysis.

### Random Sequencing

We homogenized a section of cerebrum from the alligator snapping turtle (turtle 1) and a section of the brainstem from the loggerhead turtle (turtle 3) in 450 μL 1× phosphate-buffered saline by using a QIAGEN TissueLyser LT (https://www.qiagen.com) at 35 Hz for 2 minutes with a 5-mm sterile stainless-steel bead (QIAGEN). Homogenized samples underwent viral enrichment (*14*,*15*). We extracted RNA by using Trizol LS Reagent (Thermo Fisher Scientific, https://www.thermofisher.com) and prepared the cDNA library as previously described (*16*,*17*)​, using manufacturer-suggested kits for ligation-based sequencing of amplicons (SQK-LSK110 with EXP-PBC096) and sequencing on a FLO-MIN106 R9.4.1 flow cell in a MinION Mk1B sequencing device (Oxford Nanopore Technologies, https://nanoporetech.com). The postsequencing analysis workflow followed the randomly primed, MinION-based sequencing as previously described (*16*,*17*). We first accomplished screening for potential pathogens by pairwise alignment of reads (BLAST, https://blast.ncbi.nlm.nih.gov/Blast.cgi]) to the National Center for Biotechnology Information (NCBI, https://www.ncbi.nlm.nih.gov) nonredundant nucleotide database (updated June 4, 2022) through the Georgia Advanced Computing Research Center (https://gacrc.uga.edu) by using default settings. After identifying piscichuvirus-like reads, we used a long-read aligner (Centrifuge version 1.0.4) (*18*)​ with a custom index to filter out host reads by using the green sea turtle genome (GCF_015237465.1_rCheMyd.pri.v2, accessed July 2021) ​combined with a publicly available Centrifuge index for bacteria and archaea (https://ccb.jhu.edu/software/centrifuge/manual.shtml). We mapped the remaining reads >50 nt to the closest piscichuviral genome to iteratively assemble the turtle virus genomes in Geneious Prime 2019.1.3 (https://www.geneious.com). We confirmed assembly with de novo assembly by using Flye (*19*).

We extracted RNA from turtle 2 by using an RNeasy Mini kit (QIAGEN) and generated a cDNA library by using a NEBNext Ultra RNA Library Prep Kit (Illumina, https://www.illumina.com), which we sequenced on the iSeq 100 Sequencing System (Illumina). We processed raw data to remove host reads by first running Kraken version 2 (*20*)​ against a custom database created by using the green sea turtle genome (NCBI assembly GCA_000344595, accessed March 2020). We assembled the remaining paired-end reads (742,979) by using SPAdes version 3.15.3 with default parameters (*21*). We then subjected the assembled contigs to BLASTX searches in OmicsBox version 2.0 (BioBam Bioinformatics, https://www.biobam.com) against the NCBI nonredundant protein database.

### Genome Analysis

We annotated the ORFs by using the NCBI ORFfinder (https://www.ncbi.nlm.nih.gov/orffinder) and by manually comparing them with the annotations of other piscichuviruses. We filled the gaps in the consensus sequences by PCR for MinION (turtles 1 and 3) or Sanger (turtle 2) sequencing. To predict RNA secondary structures at genomic termini, we used the Vienna package RNAfold tool (*22*).

### ISH

We used ISH to localize turtle piscichuviral mRNA in all 6 turtles with idiopathic meningoencephalomyelitis and 6 control turtles. RNAscope 2.5 HD double z-probes (Advanced Cell Diagnostics, Inc., http://acdbio.com) were designed by using the large protein gene (*L*) gene (of freshwater turtle neural virus 1 [FTuNV1] probe: 1375–2368, sea turtle neural virus 1 [STuNV1] probe: 1535–2585). We used probes targeting testudine rRNA as positive probe controls and the *Bacillus subtilis* dihydrodipicolinate reductase (DapB) gene as negative probe controls (Advanced Cell Diagnostics, Inc.). RNAscope (Advanced Cell Diagnostics, Inc.) ISH was performed by following the manufacturer’s protocols and analyzed by light microscopy.

### Phylogenetic Analyses

To infer evolutionary relationships, we phylogenetically analyzed the translated *L* ORFs from FTuNV1, 2 isolates of STuNV1, and 56 other complete jingchuvirals in MEGA X (*23*). We performed multiple sequence alignments of each coding sequence separately by using ClustalW (https://www.clustal.org) and MUSCLE (https://www.ebi.ac.uk/Tools/msa/muscle) with default settings. We based selection of the best substitution model of aligned amino acid sequences on the lowest Bayesian information criterion and Akaike scores and used the best substitution model analysis for maximum-likelihood analysis. We constructed phylogenetic analyses by using the maximum-likelihood method and the Le Gascuel matrix plus observed amino acid frequencies plus 5 discrete gamma categories distribution plus invariant sites substitution model with 500 bootstrap replicates. We used subtree-pruning-regrafting level 3 (MEGA X) for maximum-likelihood tree inference and used all gaps and missing data to construct the phylogenetic tree.

## Results

### Animal Histories and Pathologies

**Figure 2:**
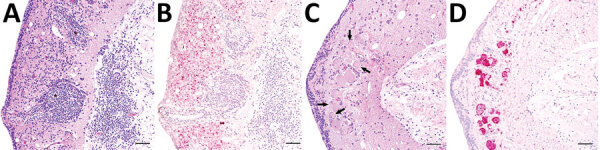
Representative tissue sections from the central nervous system of an alligator snapping turtle (*Macrochelys* sp.) with meningoencephalomyelitis, United States, 2009. A) Cerebellum; lymphoplasmacytic perivascular cuffs (asterisk) and infiltrates are widely disseminated in the gray matter and the adjacent leptomeninges. Hematoxylin and eosin stain. B) Replicate section of the same tissue shown in panel A. There is strong in situ hybridization signal (red) against freshwater turtle neural virus 1 (FTuNV1) in the cytoplasm of small neurons and glial cells throughout the gray matter and associated with the lymphoplasmacytic infiltrates. Hematoxylin counterstain. C) Optic tectum; several neurons have central chromatolysis (arrows). Hematoxylin and eosin stain. D) A replicate section of the tissue shown in panel C. Intense in situ hybridization signal (red) against FTuNV1 was within the neuronal and glial cytoplasm. Scale bars indicate 50 µm.

Six test turtles (turtles 1–6) with histories of persistent neurologic signs (e.g., weakness, lethargy, asymmetric buoyancy, circling, head tremors, cervical ventroflexion, and unresponsiveness) were collected from the southeastern United States; 5 were piscichuvirus-positive (Table 1; Figure 1). The CNSs of all turtles were grossly normal; however, those turtles had moderate to severe, multifocal to diffuse mononuclear meningoencephalomyelitis with severe lymphoplasmacytic cuffs. Most severely affected were the cerebrum, optic tectum, and cerebellum (Figure 2, panels A, C). The associated neuroparenchyma was vacuolated, and some neurons exhibited central chromatolysis (Figure 2, panel C). Subsequent results from ancillary testing (i.e., Ziehl-Neelsen staining [turtles 2–4] or PCR for herpesvirus [turtle 1] [*24*] and turtle fraservirus 1 [turtle 4] [*4*]) were negative. Turtles 1–3 were used for metagenomic sequencing; turtles 1–6, along with 6 control turtles, were used for ISH.

### Viral Genomes

#### FTuNV1/Alligator Snapping Turtle

Through reference-based alignment of all reads that passed quality filtering when BLASTN was used, we detected only a few piscichuviral-like reads, including hits to Wēnlǐng fish chu-like virus (WFClV; *Piscichuvirus wenlingense*, GenBank accession no. MG600011) and HFrV1 (GenBank accession nos. MN567051, MN567057, MN56703). Mapping filtered reads to WFClV in Geneious resulted in 1,491 piscichuviral reads and a draft FTuNV1 genome. After targeted sequencing to close gaps, we obtained a 10,781-nt complete FTuNV1 genome with at least 10 times coverage: (Table 2; isolate FTuNV1/Alligator_snapping_turtle/Florida/ST0994/2009, GenBank accession no, OQ547744).

**Table 2:** Genome comparison of piscichuviruses from 5 turtles positive for FTuNV1 or StuNV1, United States, 2009–2021, and reference sequences*

Virus	GenBank accession no.	Genome, nt	3′ UTR, nt	ORF4, nt	N, nt	G, nt	L, nt	5′ UTR, nt
FTuNV1	OQ547744	10,781	89	318	1,446	2,052	6,438	91
STuNV1 (Kemp’s ridley)	OQ547745	10,839	93	318	1,500	2,052	6,438	91
STuNV1 (Loggerhead)	OQ547746	10,839	93	318	1,500	2,052	6,438	91
GRSCV	MG600009	10,625	>59	276^‡^	1,482	1,995	6,423	>67
WFClV	MG600011	10,385	>64	225^‡^	1,344	1,929	6,348	>45
HFrV1	MN567051	10,718	79	255^‡^	1,509	1,983	6,435	204
HhCV	MW645030–2	10,858	NA	NA	1,566	1,956	6,363	NA
SxASC4	KX884439	11,270	>177	234†	1,794	2,046	6,468	>97
FMCV1	ON125109	10,991	25	396	1,395	2,178	6,615	104

*FMCV1, freshwater macrophyte associate chu-like virus 1; FTuNV1, freshwater turtle neural virus 1; G, glycoprotein; GRSCV, Guǎngdōng red-banded snake chuvirus-like; HFrV1, Herr Frank virus 1; HhCV, hardyhead chuvirus; L, large protein; N, nucleoprotein; NA, data not available; ORF, open reading frame; STuNV1, sea turtle neural virus 1; SxASC4, Sānxiá atyid shrimp virus 4; UTR, untranslated region; WFClV, Wēnlǐng fish chu-like virus. †Annotations were performed in this study because ORF4 was not originally annotated in the National Center for Biotechnology Information.

#### STuNV1/Kemp’s Ridley Turtle

 Using BLASTX, we identified 4 de novo contigs with highest similarity to HFrV1 (GenBank accession no. MN567051) and Guǎngdōng red-banded snake chuvirus-like virus (GRSCV; *Piscichuvirus lycodontis,* GenBank accession no. MG600009). After performing targeted sequencing to close gaps, we obtained a 10,839-nt complete genome (GenBank accession no. OQ547745).

#### STuNV1/Loggerhead Turtle

After initially detecting piscichuvirus-like reads by using Centrifuge (with a custom index [*16*] containing FTuNV1 and STuNV1), we identified piscichuviral reads by mapping to the Kemp’s ridley STuNV1 consensus sequence. That process resulted in 258 reads building a 10,839-nt complete genome with at least 10 times coverage, except for the first 9 bases of 5′ terminus, which had 6–9 times coverage (GenBank accession no. OQ547746.)

### Genome Comparison of Piscichuviruses

**Figure 3:**
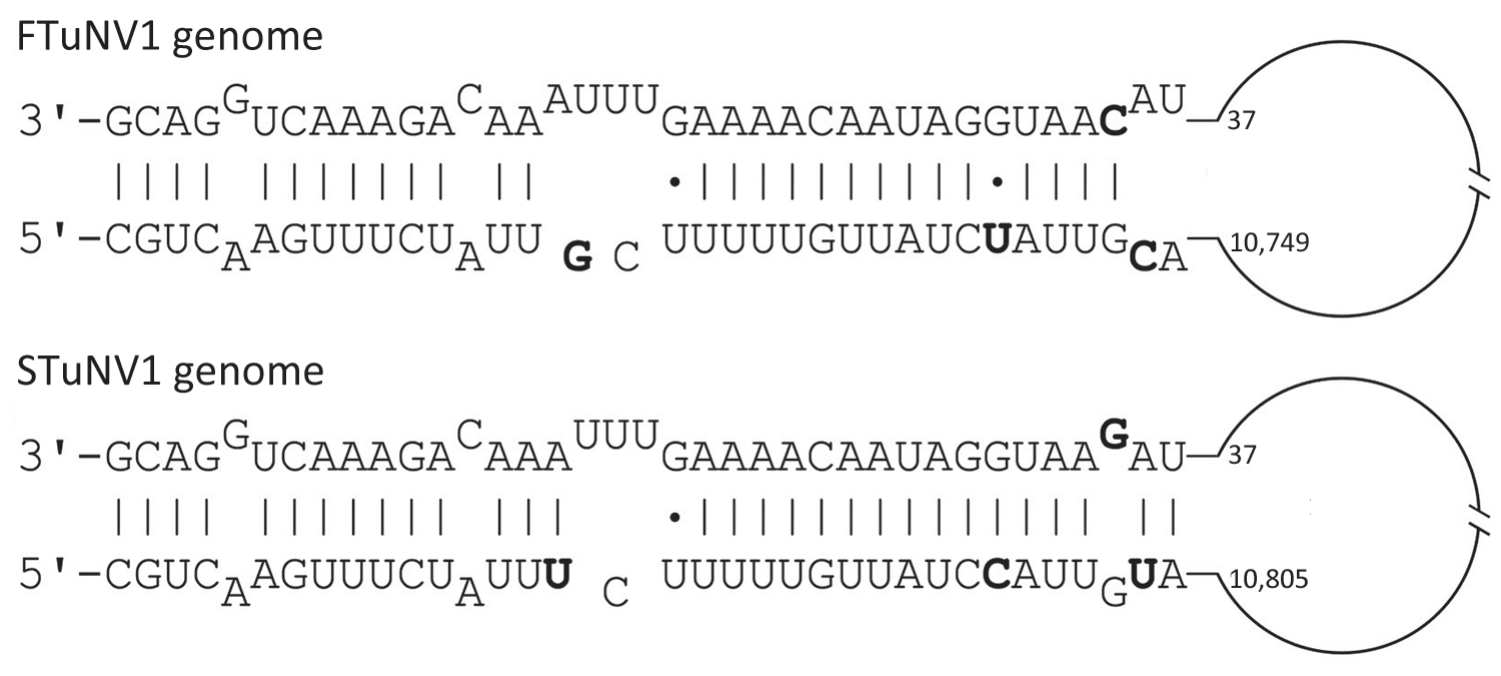
Terminal structure showing panhandle sequences for the 3′ and 5′ termini of FTuNV1 and STuNV1 genomes from piscichuvirus-infected aquatic turtles with meningoencephalomyelitis, United States, 2009–2021. Sequences were predicted by using the Vienna package RNAfold tool on Geneious (https://www.geneious.com). Offset bases indicate mispairing. Boldface bases indicate nucleotide differences between FTuNV1 and STuNV1. Dots indicate G/U pairings. The remaining genome is indicated by the loop structure (not to scale). FTuNV1, freshwater turtle neural virus 1; STuNV1, sea turtle neural virus 1.

The genomic structures of FTuNV1 and STuNV1 were linear, nonsegmented, and had the following ORF orientation: 3′-ORF4, nucleoprotein (N), glycoprotein (G), large protein (L)-5′. In addition, we identified that the genomic termini were complementary (i.e., inverted terminal repeat sequences), and in silico modeling predicted the formation of a genomic panhandle structure for each virus (Figure 3).

To taxonomically classify FTuNV1 and STuNV1, we used the recent jingchuviral taxonomic classification, which is based on the L protein amino acid identity (*21*). The percentage pairwise amino acid identities <90%, <31%, and <21% support the differentiation of jingchuvirals as novel species, genera, and families, respectively (*12*). We determined that the L protein of the STuNV1 isolates had the same predicted length (2,145 aa; Table 2) and were 99.3% identical (Appendix); thus, they were considered to be 2 isolates of a single variant. The FTuNV1 and STuNV1 L protein sequences had the same predicted length but were ≈92% identical (Appendix), which is close to the initially proposed speciation cutoff criterion. Thus, all 3 isolates are considered to be within the same new species, but FTuNV1 and STuNV1 are proposed as variants within this species.

We identified a fourth ORF4 in FTuNV1 and STuNV1. The predicted ORF4 amino acid sequence length was the same in all 3 turtle isolates (105 aa; Table 2), and the predicted amino acid sequences were identical for the 2 isolates of STuNV1. Predicted identity between FTuNV1 and STuNV1 was ≈77.36% (Appendix). Of note, we also identified putative, but unannotated, ORF4s in previously NCBI-deposited piscichuviral sequences (Table 2). Among the ORFs, ORF4 is predicted to have the most amino acid variation across piscichuviruses (Appendix). For piscichuviruses that were previously deposited in GenBank, the putative ORF4 was 225–276 nt (74–91 aa) long and 3′ prime of the N ORF (Table 2). In addition, ORF4 lacked evidence of transmembrane domains (https://services.healthtech.dtu.dk/TMHMM-2.0), signal sequences (https://services.healthtech.dtu.dk/SignalP-5.0), and N-linked glycosylation sites (https://services.healthtech.dtu.dk/NetNGlyc-1.0).

### Phylogeny of Jingchuvirals

**Figure 4:**
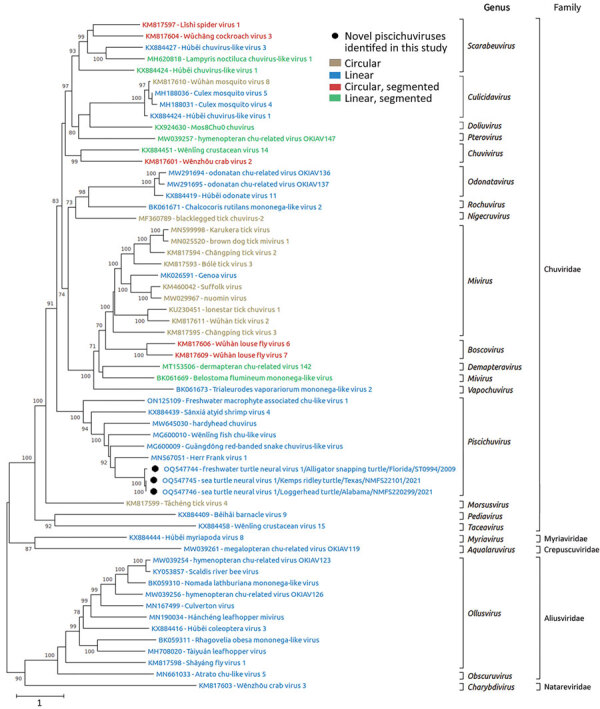
Phylogenetic analysis of jingchuviral large protein (L) amino acid sequences from piscichuvirus-infected aquatic turtles with meningoencephalomyelitis, United States, 2009–2021 (black dots), and reference sequences**.** Complete L amino acid sequences were aligned by using ClustalW (https://www.clustal.org) and refined by using MUSCLE (https://www.ebi.ac.uk/Tools/msa/muscle) with default settings. The phylogenetic analysis was performed on MEGA X (*23*) by using the maximum-likelihood method and Le Gascuel matrix plus observed amino acid frequencies plus 5 discrete gamma categories distribution with parameter of 1.0728 plus invariant sites with 0.65% sites. The substitution model was constructed with 500 bootstrap replicates. The tree is drawn to scale; bootstrap values are measured in the number of substitutions per site. This analysis included 59 aa sequences. Sequences are color coded based on their genomic structure.

Phylogenetic analysis of the predicted L protein amino acid sequences from 59 chuvirids demonstrated that FTuNV1 and both STuNV1 isolates clustered with other piscichuviruses; bootstrap value was 100%. All piscichuviruses detected from reptiles form a single branch with a 92% bootstrap value (Figure 4). Similarly, all piscichuviruses detected from vertebrates form a single branch with a 100% bootstrap value (Figure 4).

### Localization of FTuNV1 and STuNV1 Nucleic Acid

To more definitively associate piscichuviral infection with the clinical and histopathologic findings, we conducted RNAscope ISH on all 3 isolates that were positive for piscichuvirus by sequencing (from turtles 1–3), all 3 of which demonstrated viral RNA within inflamed areas of the CNS (Figure 2, panels C, D). ISH testing of 3 additional turtles (turtles 4–6), in which no sequencing was performed indicated that 2 of the 3 were positive. Thus, 5 of 6 cases that were originally considered idiopathic were proposed to be associated with piscichuvirus (Table 1).

ISH demonstrated disseminated, strong, punctate reactivity for piscichuviral RNA in areas of inflammation throughout the CNS. Piscichuviral RNA was detected predominantly in the gray matter (Figure 2, panel B), most notably within the cytoplasm of large neurons (often chromatolytic), small neurons, glial cells, but occasionally in ependymal cells with subtle intensity (Figure 2, panel D). Testing of nonneural tissues of turtles 1 (tested for FTuNV1 nucleic acid) and 2 (tested for STuNV1 nucleic acid) did not demonstrate viral mRNA staining.

None of the control brain tissues demonstrated viral RNA staining. The host control probe reacted appropriately in all tissues that were virus negative by ISH. Neither probe detected the other variants.

## Discussion

According to the current criteria of using the L protein amino acid sequence similarity for jingchuviral speciation (*12*), these novel turtle jingchuvirals represent a new species within the genus *Piscichuvirus.* However, the original speciation criteria might need to be revisited to determine if the 2 variants (i.e., FTuNV1 and STuNV1) actually represent 2 different piscichuviral species given their relative dissimilarity (92%) and host differences. For example, although sea turtles are known to forage within tidal areas of rivers (*25*) ​and freshwater turtles, including alligator snapping turtles, are occasionally documented in estuarine and marine waters (*26*), those ecosystems are relatively nonoverlapping. As additional studies reveal more about the diversity within and between chuvirids, their evolutionary timeline, and their host restrictions, it is foreseeable that these 2 turtle variants might ultimately be divided into at least 2 species (e.g., freshwater [chelydroid] and marine [chelonioid] turtle).

The predicted terminal panhandle structures of the turtle neural virus genomes are similar to those of many other viruses of phylum Negarnaviricota, including bunyavirals, orthomyxovirids, paramyxovirids, and rhabdovirids (*27*–*29*) but have not been reported for jingchuvirals. In orthomyxovirids, those structures serve as promoters for transcription (*30*–*32*), but by creating double-stranded RNA, they also induce the antiviral activity of retinoic acid-inducible gene I. Although the biological effect of this structure remains to be determined, the putative panhandle-forming untranslated regions could be used for the in silico identification of genomic ends in chuvirids discovered in the future through metagenomics and might provide more insight into the development of genomic structure diversity within Chuviridae.

Recent viral zoonoses (e.g., severe acute respiratory syndrome, Ebola virus disease, AIDS) demonstrate that wildlife species can be reservoirs (*33*); thus, it is imperative to fully document the repertoire of viruses in wildlife and their association with disease. Chuvirids are the only jingchuvirals that have been identified in vertebrates, including fish and reptiles (*9*,*13*). However, any associations with the disease have been weak and lacked in situ viral localization (*13*). Our study successfully localized chuvirid mRNA within the areas of inflammation in multiple individuals across 3 turtle species from 2 different ecosystems. Because sequence-based approaches have become a common platform for disease detection and characterization, modifications to Koch’s postulates have been proposed to establish the causal association of a novel agent in which Koch’s postulates cannot be fulfilled (e.g., infection of novel agents in endangered species and a likely irreversible condition [meningoencephalomyelitis]) (*34*). The 2 turtle piscichuviral variants have met 3 of the 7 proposed criteria: 1) FTuNV1 and STuNV1 nucleic acid sequences were detected in diseased tissues, 2) no nucleic acid sequence was detected in tissues without disease, and 3) infection was confirmed at the cellular level via ISH. Although further research on this disease is required to verify reproducibility and to identify similar biological properties in other hosts, those findings strongly suggest that FTuNV1 and STuNV1 are a cause of severe mononuclear meningoencephalomyelitis in aquatic turtles in multiple ecosystems throughout the southeastern United States. The identification of closely related chuvirids in other reptiles and fish suggests that chuvirids should be considered as potential emerging viruses in at least fish and reptiles, if not mammals. Further surveillance is needed to better determine the effect of chuvirids on those and other turtles.

All of the turtle species in which a chuvirid was found are considered imperiled. Affected turtles included mature adults, which are especially vital to the stability and recovery of turtle populations (*35*). Of note, all 3 turtles with known body condition scores (turtles 1, 4, and 5) were in good nutritional condition at the time of death, and all infected turtles lacked any predisposing conditions that would increase susceptibility to virus infection. In addition, 2 STuNV1-infected loggerhead turtles (turtles 4 and 5) were stranded ≈1 month apart within the same geographic region. The potential to infect and cause disease in relatively healthy individuals and the identification of multiple diseased turtles from the same areas and time indicate a serious wildlife health concern. In addition, an observation associated with 1 of the cases reported here raises the possibility of human-mediated pathogen pollution. The genus *Macrochelys* is proposed to include either 2 or 3 species (*36*,*37*). The alligator snapping turtle infected by FTuNV1 was morphologically consistent with the more western member(s) of the genus, either *M*. *temminckii* or *M*. *apalachicolae*, neither of which should be located where that turtle was found. Thus, the discovery of that turtle outside of its natural range suggests that it may have been transported and released. Future studies are needed to understand the diversity and prevalence of chuvirids among turtles, the pathogenesis of chuvirid infections, and the effects of environment on disease susceptibility.

In summary, we identified 2 variants of a new piscichuviral species in 5 aquatic turtles that died of idiopathic meningoencephalomyelitis. FTuNV1 and STuNV1 most likely cause lymphocytic meningoencephalomyelitis in multiple aquatic turtle species. 

Appendix Additional information for study of piscichuvirus-associated severe meningoencephalomyelitis in aquatic turtles, United States, 2009–2021.

## References

[1] Senko JF, Burgher KM, Del Mar Mancha-Cisneros M, Godley BJ, Kinan-Kelly I, Fox T, et al. Global patterns of illegal marine turtle exploitation. Glob Change Biol. 2022;28:6509–23. 10.1111/gcb.16378 36069207

[2] Rhodin AGJ, Iverson JB, Bour R, Fritz U, Georges A, Shaffer HB, et al.; Turtle Taxonomy Working Group. Turtles of the world: annotated checklist and atlas of taxonomy, synonymy, distribution, and conservation status. In: Rhodin AGJ, Iverson JB, van Dijk PP, Stanford CB, Goode EV, Buhlmann, KA, et al., editors. Conservation Biology of Freshwater Turtles and Tortoises: a Compilation Project of the IUCN/SSC Tortoise and Freshwater Turtle Specialist Group. 9th ed. Chelonian Research Monographs. Rochester (NY): Mercury Print Productions. 2021;8:1–472.

[3] Greenblatt RJ, Work TM, Dutton P, Sutton CA, Spraker TR, Casey RN, et al. Geographic variation in marine turtle fibropapillomatosis. J Zoo Wildl Med. 2005;36:527–30. 10.1638/04-051.1 17312778

[4] Waltzek TB, Stacy BA, Ossiboff RJ, Stacy NI, Fraser WA, Yan A, et al. A novel group of negative-sense RNA viruses associated with epizootics in managed and free-ranging freshwater turtles in Florida, USA. PLoS Pathog. 2022;18:e1010258. 10.1371/journal.ppat.1010258 35275967 PMC8916662

[5] Harding EF, Russo AG, Yan GJH, Mercer LK, White PA. Revealing the uncharacterised diversity of amphibian and reptile viruses. ISME Commun. 2022;2:95. 10.1038/s43705-022-00180-x 37938670 PMC9723728

[6] Li CX, Shi M, Tian JH, Lin XD, Kang YJ, Chen LJ, et al. Unprecedented genomic diversity of RNA viruses in arthropods reveals the ancestry of negative-sense RNA viruses. eLife. 2015;4:1–26. 10.7554/eLife.05378 25633976 PMC4384744

[7] Han X, Wang H, Wu N, Liu W, Cao M, Wang X. Leafhopper *Psammotettix alienus* hosts chuviruses with different genomic structures. Virus Res. 2020;285:197992. 10.1016/j.virusres.2020.197992 32371097

[8] Shi M, Lin XD, Tian JH, Chen LJ, Chen X, Li CX, et al. Redefining the invertebrate RNA virosphere. Nature. 2016;540:539–43. 10.1038/nature20167 27880757

[9] Shi M, Lin XD, Chen X, Tian JH, Chen LJ, Li K, et al. The evolutionary history of vertebrate RNA viruses. Nature. 2018;556:197–202. 10.1038/s41586-018-0012-7 29618816

[10] Hahn MA, Rosario K, Lucas P, Dheilly NM. Characterization of viruses in a tapeworm: phylogenetic position, vertical transmission, and transmission to the parasitized host. ISME J. 2020;14:1755–67. 10.1038/s41396-020-0642-2 32286546 PMC7305300

[11] Dezordi FZ, Vasconcelos CRDS, Rezende AM, Wallau GL. In and outs of *Chuviridae* endogenous viral elements: origin of a potentially new retrovirus and signature of ancient and ongoing arms race in mosquito genomes. Front Genet. 2020;11:542437. 10.3389/fgene.2020.542437 33193616 PMC7642597

[12] Di Paola N, Dheilly NM, Junglen S, Paraskevopoulou S, Postler TS, Shi M, et al. *Jingchuvirales*: a new taxonomical framework for a rapidly expanding order of unusual monjiviricete viruses broadly distributed among arthropod subphyla. Appl Environ Microbiol. 2022;88:e0195421. 10.1128/aem.01954-21 35108077 PMC8939347

[13] Argenta FF, Hepojoki J, Smura T, Szirovicza L, Hammerschmitt ME, Driemeier D, et al. Identification of reptarenaviruses, hartmaniviruses and a novel chuvirus in captive Brazilian native boa constrictors with boid inclusion body disease. J Virol. 2020;94:1–19. 10.1128/JVI.00001-20 32238580 PMC7269426

[14] Conceição-Neto N, Yinda KC, Van Ranst M, Matthijnssens J. NetoVIR: modular approach to customize sample preparation procedures for viral metagenomics. In: Moya A, Pérez Brocal V, editors. The Human Virome In Molecular Biology. Totowa (NJ): Humana Press Inc.; 2018. p. 85–95. 10.1007/978-1-4939-8682-8_7 30128991

[15] Vibin J, Chamings A, Collier F, Klaassen M, Nelson TM, Alexandersen S. Metagenomics detection and characterisation of viruses in faecal samples from Australian wild birds. Sci Rep. 2018;8:8686. 10.1038/s41598-018-26851-1 29875375 PMC5989203

[16] Young KT, Stephens JQ, Poulson RL, Stallknecht DE, Dimitrov KM, Butt SL, et al. Putative novel avian paramyxovirus (AMPV) and reidentification of APMV-2 and APMV-6 to the species level based on wild bird surveillance (United States, 2016–2018). Appl Environ Microbiol. 2022;88:e0046622. 10.1128/aem.00466-22 35612300 PMC9195946

[17] Young KT, Lahmers KK, Sellers HS, Stallknecht DE, Poulson RL, Saliki JT, et al. Randomly primed, strand-switching, MinION-based sequencing for the detection and characterization of cultured RNA viruses. J Vet Diagn Invest. 2021;33:202–15. 10.1177/1040638720981019 33357075 PMC7953086

[18] Kim D, Song L, Breitwieser FP, Salzberg SL. Centrifuge: rapid and sensitive classification of metagenomic sequences. Genome Res. 2016;26:1721–9. 10.1101/gr.210641.116 27852649 PMC5131823

[19] Kolmogorov M, Yuan J, Lin Y, Pevzner PA. Assembly of long, error-prone reads using repeat graphs. Nat Biotechnol. 2019;37:540–6. 10.1038/s41587-019-0072-8 30936562

[20] Wood DE, Lu J, Langmead B. Improved metagenomic analysis with Kraken 2. Genome Biol. 2019;20:257. 10.1186/s13059-019-1891-0 31779668 PMC6883579

[21] Bankevich A, Nurk S, Antipov D, Gurevich AA, Dvorkin M, Kulikov AS, et al. SPAdes: a new genome assembly algorithm and its applications to single-cell sequencing. J Comput Biol. 2012;19:455–77. 10.1089/cmb.2012.0021 22506599 PMC3342519

[22] Hofacker IL. Vienna RNA secondary structure server. Nucleic Acids Res. 2003;31:3429–31. 10.1093/nar/gkg599 12824340 PMC169005

[23] Kumar S, Stecher G, Li M, Knyaz C, Tamura K. MEGA X: Molecular Evolutionary Genetics Analysis across computing platforms. Mol Biol Evol. 2018;35:1547–9. 10.1093/molbev/msy096 29722887 PMC5967553

[24] VanDevanter DR, Warrener P, Bennett L, Schultz ER, Coulter S, Garber RL, et al. Detection and analysis of diverse herpesviral species by consensus primer PCR. J Clin Microbiol. 1996;34:1666–71. 10.1128/jcm.34.7.1666-1671.1996 8784566 PMC229091

[25] Byles RA. Behavior and ecology of sea turtles from Chesapeake Bay, Behavior and ecology of sea turtles from Chesapeake Bay, Virginia [dissertation]. Williamsburg (VA): College of William and Mary; 1988.

[26] Jackson GJ Jr, Ross A. The occurrence of barnacles on the alligator snapping turtle, *Macrochelys temminckii* (Troost). J Herpetol. 1971;5:188–9. 10.2307/1562744

[27] Obijeski JF, McCauley J, Skehel JJ. Nucleotide sequences at the terminal of La Crosse virus RNAs. Nucleic Acids Res. 1980;8:2431–8. 10.1093/nar/8.11.2431 7443511 PMC324091

[28] Hsu MT, Parvin JD, Gupta S, Krystal M, Palese P. Genomic RNAs of influenza viruses are held in a circular conformation in virions and in infected cells by a terminal panhandle. Proc Natl Acad Sci U S A. 1987;84:8140–4. 10.1073/pnas.84.22.8140 2446318 PMC299494

[29] Auperin DD, Romanowski V, Galinski M, Bishop DH. Sequencing studies of pichinde arenavirus S RNA indicate a novel coding strategy, an ambisense viral S RNA. J Virol. 1984;52:897–904. 10.1128/jvi.52.3.897-904.1984 6492264 PMC254611

[30] Fodor E, Pritlove DC, Brownlee GG. The influenza virus panhandle is involved in the initiation of transcription. J Virol. 1994;68:4092–6. 10.1128/jvi.68.6.4092-4096.1994 8189550 PMC236924

[31] Neumann G, Hobom G. Mutational analysis of influenza virus promoter elements in vivo. J Gen Virol. 1995;76:1709–17. 10.1099/0022-1317-76-7-1709 9049376

[32] Flick R, Neumann G, Hoffmann E, Neumeier E, Hobom G. Promoter elements in the influenza vRNA terminal structure. RNA. 1996;2:1046–57. 8849780 PMC1369436

[33] Keatts LO, Robards M, Olson SH, Hueffer K, Insley SJ, Joly DO, et al. Implications of zoonoses from hunting and use of wildlife in North American arctic and boreal biomes: pandemic potential, monitoring, and mitigation. Front Public Health. 2021;9:627654. 10.3389/fpubh.2021.627654 34026707 PMC8131663

[34] Fredricks DN, Relman DA. Sequence-based identification of microbial pathogens: a reconsideration of Koch’s postulates. Clin Microbiol Rev. 1996;9:18–33. 10.1128/CMR.9.1.18 8665474 PMC172879

[35] Heppell SS. Application of life-history theory and population model analysis to turtle conservation. Copeia. 1998;1998:367–75. 10.2307/1447430

[36] Thomas TM, Granatosky MC, Bourque JR, Krysko KL, Moler PE, Gamble T, et al. Taxonomic assessment of Alligator Snapping Turtles (Chelydridae: *Macrochelys*), with the description of two new species from the southeastern United States. Zootaxa. 2014;3786:141–65. 10.11646/zootaxa.3786.2.4 24869532

[37] Folt B, Guyer C. Evaluating recent taxonomic changes for alligator snapping turtles (Testudines: Chelydridae). Zootaxa. 2015;3947:447–50. 10.11646/zootaxa.3947.3.11 25947748

